# Folic acid supplementation, dietary folate intake during pregnancy and risk for spontaneous preterm delivery: a prospective observational cohort study

**DOI:** 10.1186/s12884-014-0375-1

**Published:** 2014-11-02

**Authors:** Verena Sengpiel, Jonas Bacelis, Ronny Myhre, Solveig Myking, Aase Serine Devold Pay, Margaretha Haugen, Anne-Lise Brantsæter, Helle Margrete Meltzer, Roy Miodini Nilsen, Per Magnus, Stein Emil Vollset, Staffan Nilsson, Bo Jacobsson

**Affiliations:** Department of Obstetrics and Gynaecology, Sahlgrenska Academy, Sahlgrenska University Hospital/Östra, Göteborg, SE-416 85 Sweden; Department of Genes and Environment, Division of Epidemiology, Norwegian Institute of Public Health, Nydalen, P.O. Box 4404, Oslo, NO-0403 Norway; Department of Obstetrics and Gynecology, Women and Children’s Division, Oslo University Hospital, Nydalen, P.O. Box 4950, Oslo, NO-0424 Norway; Department of Exposure and Risk Assessment, Division of Environmental Medicine, Norwegian Institute of Public Health, Nydalen, P.O. Box 4403, Oslo, NO-0403 Norway; Department of Global Public Health and Primary Care, University of Bergen, Bergen, NO-5018 Norway; Division of Epidemiology, Norwegian Institute of Public Health, P.O. Box 4403, Oslo, NO-0403 Norway; Norwegian Institute of Public Health and University of Bergen, Kalfarveien 31, Bergen, NO-5018 Norway; Mathematical Sciences, Chalmers University of Technology, Göteborg, SE-412 96 Sweden

**Keywords:** Pregnancy, Preterm delivery, Preterm birth, Gestational length, Folate, Folic acid supplementation

## Abstract

**Background:**

Health authorities in numerous countries recommend periconceptional folic acid supplementation to prevent neural tube defects. The objective of this study was to examine the association of dietary folate intake and folic acid supplementation during different periods of pregnancy with the risk of spontaneous preterm delivery (PTD).

**Methods:**

The Norwegian Mother and Child Cohort Study is a population-based prospective cohort study. A total of 66,014 women with singleton pregnancies resulting in live births in 2002–2009 were included. Folic acid supplementation was self-reported from 26 weeks before pregnancy until pregnancy week 24. At gestational week 22, the women completed a food frequency questionnaire, which allowed the calculation of their average total folate intake from foods and supplements for the first 4–5 months of pregnancy. Spontaneous PTD was defined as the spontaneous onset of delivery between weeks 22^+0^ and 36^+6^ (n = 1,755).

**Results:**

The median total folate intake was 313 μg/d (interquartile range IQR 167–558) in the overall population and 530 μg/d (IQR 355–636) in the supplement users. Eighty-five percent reported any folic acid supplementation from <8 weeks before to 24 weeks after conception while only 44% initiated folic acid supplementation before pregnancy. Cox regression analysis showed that the amount of dietary folate intake (hazard ratio HR 1.00; confidence interval 95% CI 0.61-1.65) and supplemental folate intake (HR 1.00; CI 1.00-1.00) was not significantly associated with the risk of PTD. The initiation of folic acid supplementation more than 8 weeks before conception was associated with an increased risk for spontaneous PTD (HR 1.18; CI 1.05-1.32) compared to no folic acid supplementation preconception. There was no significant association with PTD when supplementation was initiated within 8 weeks preconception (HR 0.99; CI 0.87-1.13). All analyses were adjusted for maternal characteristics and socioeconomic, health and dietary variables.

**Conclusions:**

Our findings do not support a protective effect of dietary folate intake or folic acid supplementation on spontaneous PTD. Preconceptional folic acid supplementation starting more than 8 weeks before conception was associated with an increased risk of spontaneous PTD. These results require further investigation before discussing an expansion of folic acid supplementation guidelines.

**Electronic supplementary material:**

The online version of this article (doi:10.1186/s12884-014-0375-1) contains supplementary material, which is available to authorized users.

## Background

Folate is a B-vitamin essential for one-carbon metabolism and takes part in amino acid metabolism as well as DNA synthesis, repair and methylation [1,2]. Women are especially susceptible to folate deficiency during pregnancy, which is a period of rapid fetal growth, organ differentiation and high rates of cell division [1,3,4]. Since the 1950s, folic acid supplementation has been known to prevent megaloblastic anemia during pregnancy [5]. In the 1990s, large randomized trials demonstrated that periconceptional folic acid supplementation can prevent neural tube defects (NTDs) in the newborn infant [6-8]. Today, national health authorities in many countries recommend periconceptional folic acid supplementation, and some countries have introduced mandatory folate fortification of foods [1,3,4,9,10]. In Norway, folic acid supplementation of 400 μg/d is recommended from the time of planning a pregnancy to gestational week 12 [2,11], as is a daily folate intake of 500 μg/d. This is in line with the Nordic Nutrition Recommendations [2].

Maternal folate status has also been associated with other adverse pregnancy outcomes such as preeclampsia, malformations such as orofacial clefts, spontaneous abortion, fetal death, fetal growth restriction and preterm delivery (PTD), although these results still remain inconclusive [1].

PTD, defined by the World Health Organization (WHO) as birth occurring before 37 weeks of gestation, is considered a major global health problem and is strongly associated with neonatal mortality as well as short- and long-term morbidity [12-14]. Spontaneous PTD is a common, complex condition with a prevalence of approximately 7% in the Norwegian population [15]. However, the effect of any single environmental factor is difficult to measure without large-scale studies [15]. Modern obstetrics are still not able to predict, prevent or treat PTD [16]. Progesterone substitution, the only promising intervention identified to date, has been shown to reduce the chance of spontaneous PTD in high-risk pregnancies, but such cases account for only a small proportion of all pregnancies [17,18].

In the past decade, some observational studies have found that folic acid supplementation reduces the risk of PTD [19-22]. In some studies, this effect has been documented with an extended folic acid supplementation scheme or dosage compared with schemes based on NTD prevention, e.g., preconceptional folic acid supplementation for one year or longer [21] or third-trimester folic acid supplementation [22]. A study based on the previous Cochrane review and data of one of the largest randomized controlled trials (RCT), a recent meta-analysis of all published RCTs, as well as the recent Cochrane review based on 3 controlled trials could not confirm any effect of the maternal folate status on the gestational length or the risk of spontaneous PTD [23-25]. The comparability and generalizability of these earlier studies, which focused on the association of folate status and folic acid supplementation with pregnancy outcome, is limited because folic acid supplementation was assessed without considering other folate sources, the study populations had different levels of dietary folate intake, inadequate sample sizes, limited adjustment for important confounders, and/or retrospective study designs with folate data collection only after delivery [1,4]. Although PTD is a heterogeneous pregnancy outcome with different etiologies (early vs. late or iatrogenic vs. spontaneous), previous studies have mostly treated PTD as one entity, obscuring the differences in risk among PTD subtypes [26,27].

The Norwegian Mother and Child Cohort Study (MoBa) can meet a number of these challenges in study design, a requirement for addressing the inconsistencies in the field. MoBa includes more than 106,000 pregnancies, enabling the investigation of common complex pregnancy outcomes such as PTD. A detailed prospective assessment of folic acid supplementation starting from 6 months before conception throughout pregnancy, data regarding dietary folate intake and comprehensive information about lifestyle habits, health and socioeconomic status provide a unique opportunity to study the association between folate intake and PTD. For example, the effect of folic acid supplementation can be compared between women with low and high dietary folate intakes. By taking into account the amount of dietary folate and folic acid supplementation during different periods of pregnancy, it might be possible to define the folic acid supplementation scheme most likely to affect PTD risk.

The aim of this study was to examine the association of maternal folate intake from both supplemental and dietary sources with the risk of spontaneous PTD, with sub-analyses of early and late spontaneous PTD. The association of folic acid supplementation with PTD was studied in a stratified sample of women with low and high dietary folate intakes (</≥170 μg/d).

## Methods

### Study population

The dataset is part of the MoBa cohort, initiated by and maintained at the Norwegian Institute of Public Health [15]. In brief, MoBa is a nation-wide pregnancy cohort that has included more than 106,000 pregnancies in the years from 1999 to 2009. The women were recruited through a postal invitation in connection with a routine ultrasound examination offered to all pregnant women in Norway approximately 17 weeks of gestation. Overall, 38.5% of the invited women participated. They were asked to fill in questionnaires focused on overall health status, lifestyle behavior and diet at gestational weeks 15–17 (Q1) and 30 (Q3). At week 22, they completed a food frequency questionnaire (FFQ). All questionnaires are available from the homepage of the Norwegian Institute of Public Health [28]. The present study used data from version 6 of the quality-assured data files made available for research in 2011. The MoBa database is linked to pregnancy and birth records from the Medical Birth Registry of Norway (MBRN) [29]. Informed written consent was obtained from each participant. The Regional Committee for Medical Research and the Norwegian Data Inspectorate approved the study.

Of 106,707 pregnancies included in the MoBa version 6, 103,921 pregnancies resulted in live-born singletons. Complete data for all 3 questionnaires including information about folate intake were available for 81,329 pregnancies. Women reporting improbable energy intakes of <4.5 MJ or >20 MJ were excluded [30], leaving 80,056 pregnancies. After exclusion of women with prepregnancy diabetes mellitus, chronic hypertension, chronic kidney disease, epilepsia, rheumatoid arthritis and those that underwent in-vitro fertilization, 76,298 pregnancies were included in the study. Pregnancies with a gestational length of <22^+0^ or >42^+6^ weeks were excluded from further analysis, leaving 75,873 remaining. If a woman participated for more than one pregnancy, only her first pregnancy enrolled was included, leaving 66,014 pregnancies for analyses.

### Outcome

The gestational age in days was determined with a second-trimester ultrasound in 98.4% of the pregnancies and was based on the last menstrual period in the remaining cases. Spontaneous PTD was defined as birth after preterm labor or pre-labor rupture of the membranes between 22^+0^ and 36^+6^ weeks. To distinguish between early and late spontaneous PTDs, we used the following gestational time-windows: early (22^+0^-33^+6^) and late (34^+0^-36^+6^) PTD.

### Exposure

#### Amount of folate intake

The amount of folate intake was calculated from the MoBa FFQ, a semi-quantitative questionnaire designed to yield information regarding the dietary habits and intake of dietary supplements during the first 5 months of pregnancy. The questionnaire data were read optically, and the nutrient and energy intakes were calculated using FoodCalc [31] and the Norwegian Food Composition Table [32]. For the calculation of nutrients in the dietary supplements, an Access database containing the nutrient values of more than 1,000 dietary supplements was created and continuously updated using Microsoft Office 2003 software. Dietary supplements commonly sold in Norway were registered based on information provided by the respective manufacturer, whereas nutritional information concerning dietary supplements bought from the Internet or abroad were obtained from the manufacturer’s or supplier’s homepage. A data program connected to the Access database read all food supplements recorded by the MoBa participants. The process of extracting dietary and supplement data is described in detail elsewhere [33,34].

Dietary folate was defined as 60% of the reported folate intake from foods, as only approximately 60% may be biologically accessible in comparison to that from the synthetic folic acid in supplements [1,3]. The total folate intake was thus calculated as supplemental folic acid +0.6 × folate intake from foods.

The daily folate intake was also categorized into 4 groups: <170 μg/d (corresponding to the earlier WHO recommendation for all women to prevent anemia [10,35]), 170–500 μg/d (corresponding to the current Nordic Nutrition Recommendations for pregnant women for the prevention of NTDs [2]), 500–1000 μg/d (corresponding to the tolerable upper limit for folic acid [2]) and >1000 μg/d.

#### Timing of folic acid supplementation

The women reported their folic acid supplement use from 26 weeks before conception until gestational week 24 in 4-week intervals, including the frequency of supplementation. A woman was defined as a folic acid supplement user if she reported any use of folic acid – containing supplements for more than once a week in a registered 4-week period. Folic acid may be consumed either in the form of a folic acid supplement alone or as part of multivitamins. The most commonly used folic acid supplements for pregnant women in Norway contain 400 μg of folic acid, while the most commonly used multivitamin supplements contain 200 μg of folic acid. For this study, the start of folic acid supplementation was categorized as start during 26–9 weeks before conception, start during 8–0 weeks before conception, and no preconceptional folic acid supplementation.

### Covariates

Covariates were chosen a priori based on the available literature. Information regarding the maternal age at delivery as well as the child’s sex is available from the MBRN. Parity was based on data from both the MoBa and MBRN and based on the number of previous pregnancies of ≥22^+0^ weeks’ duration. Marital status was defined as either married/cohabitant or not. The self-reported pre-pregnancy heights and weights were used to calculate the pre-pregnancy body mass index (BMI) and were grouped according to the WHO classification as underweight (<18.5 kg/m^2^), normal (18.5-24.9 kg/m^2^), overweight (25.0-29.9 kg/m^2^) and obese (≥30.0 kg/m^2^). Maternal education was categorized as ≤12 y, 13–16 y and ≥17 y. The history of previous PTD before 37^+0^ weeks of gestation, and the history of spontaneous abortion, as registered in the MBRN, were taken into account as dichotomous variables in regression models. Women reported smoking habits during pregnancy in Q1 and were categorized as non-smokers, occasional or daily smokers. The alcohol intake from different sources was self-reported in the FFQ (glasses/d, week or month) and calculated as g/d. The household income was classified as both the participant and her partner having <300,000 Norwegian Kroner (NOK)/y, as either the participant or her partner having ≥300,000 NOK/y or as the participant and her partner both having ≥300,000 NOK/y. Vitamin A supplementation was registered and categorized in the same manner as described for folic acid. These variables were used as a proxy for multivitamin supplementation, as there are no products on the Norwegian market containing vitamin A alone, and vitamin A is part of all common multivitamin preparations. In MoBa more than 99% of the participants are of Caucasian ethnicity; hence, ethnicity was not a relevant confounder.

### Statistical methods

All statistical analyses were performed using IBM SPSS Statistics 22 and R 2.13.1 software. Total dietary folate intake from foods and supplements (median (IQR)) in relation to the maternal characteristics was studied with the Kruskal-Wallis test. The start of folic acid supplementation in relation to the maternal characteristics was studied with Pearson’s chi-squared test. The association of total dietary folate intake and spontaneous PTD was estimated as a hazard ratio (HR) with a 95% confidence interval (CI) by using Cox regression both in an unadjusted model and adjusted for the above-mentioned covariates. In these models, the event was defined as a spontaneous PTD; all iatrogenic deliveries and deliveries after the preterm (≥37^+0^ weeks) or early preterm (≥34^+0^ weeks) period were censored. The proportional hazards assumption was investigated by testing and inspecting scaled Schoenfeld residuals using R function cox.zph [36]. Statistical significance was assumed for 2-sided p-values of <0.05.

## Results

### Folate intake and folic acid supplementation in the study population

The median total dietary folate intake during the first five months of pregnancy and start of folic acid supplementation according to maternal characteristics are presented in Table 1. Dietary folate intake was highest in women aged 25–29 years, in women who did not smoke, had low or normal BMI (18.5-24.9 kg/m^2^), who were having their first child, were married/cohabitant, who had higher education levels and family incomes. Women having experienced PTD had significantly lower total folate intakes. While women with a history of spontaneous abortion had more often started folic acid supplementation early, no comparable pattern in women with a history of PTD was found (Table 1).

**Table 1:** **Folate variables and maternal characteristics**

				**Total folate intake (μg/d)**			**Initiation of preconceptional folic acid supplementation, n (%)**						
		**n**	**(%)**	**Median (IQR)**		**p** ^**1**^	**>8 w**		**0-8 w**		**No**		**p** ^**2**^
Total		66014	(100)	313	(167–558)		16023	(24)	12877	(20)	37114	(56)	
	<25	7561	(12)	258	(154–528)		849	(11)	942	(13)	5770	(76)	
Maternal age in years	25-29	22672	(34)	324	(170–562)	<0.0001	5121	(23)	4821	(21)	12730	(56)	<0.0001
	30-34	27993	(42)	319	(168–562)		7726	(28)	5894	(21)	14373	(51)	
	>34	7788	(12)	315	(170–562)		2327	(30)	1220	(16)	4241	(55)	
Pre-pregnancy BMI in kg/m^2^	<18.5	19\31	(3)	343	(179–575)		432	(22)	351	(18)	1148	(60)	
	18.5-24.9	42611	(65)	328	(173–566)	<0.0001	10773	(25)	8637	(20)	23201	(54)	<0.0001
	25-30	13839	(21)	289	(160–548)		3228	(23)	2651	(19)	7960	(58)	
	≥30	5970	(9)	268	(151–533)		1305	(22)	953	(16)	3712	(62)	
	Missing	1663	(3)	250	(156–517)		285	(17)	285	(17)	1093	(66)	
	0	34825	(53)	355	(180–580)		9324	(27)	6480	(19)	19021	(55)	
	1	20028	(30)	281	(159–545)	<0.0001	4528	(23)	4531	(23)	10969	(55)	<0.0001
Parity	2	9005	(14)	245	(156–517)		1787	(20)	1570	(17)	5648	(63)	
	3+	2101	(3)	224	(149–451)		370	(18)	285	(14)	1446	(69)	
	Missing	55	(0.1)	353	(166–566)		14	(26)	11	(20)	30	(55)	
Marita status	Yes	63518	(96)	315	(168–559)	<0.0001	15714	(25)	12661	(20)	35143	(55)	<0.0001
	No	2496	(4)	266	(158–531)		309	(12)	216	(9)	1971	(79)	
Maternal education in years	<13	20198	(31)	244	(150–515)		3263	(16)	2886	(14)	14049	(70)	
	13 - 16	27606	(42)	338	(172–567)	<0.0001	7118	(26)	5849	(21)	14639	(53)	<0.0001
	>16	16838	(26)	374	(187–581)		5340	(32)	3916	(23)	7582	(45)	
	Missing	1372	(2)	283	(161–550)		302	(22)	226	(17)	844	(62)	
History of preterm delivery	No	63698	(97)	314	(168–559)	<0.0001	15510	(24)	12411	(20)	35777	(56)	0.047
	Yes	2205	(3)	276	(158–539)		487	(22)	435	(20)	1283	(58)	
	Missing	111	(0.2)	297	(175–560)		26	(23)	31	(28)	54	(49)	
History of abortion	No	45164	(68)	315	(168–559)	0.15	10262	(23)	8930	(20)	25972	(58)	<0.0001
	Yes	12418	(19)	308	(166–558)		3932	(32)	2296	(19)	6190	(50)	
	Missing	8432	(13)	308	(165–557)		1829	(22)	1651	(20)	4952	(59)	
	Never	60452	(92)	322	(170–563)		15339	(25)	12271	(20)	32842	(54)	
Smoking habits	Occasionally	1741	(3)	261	(158–511)	<0.0001	243	(14)	226	(13)	1272	(73)	<0.0001
	Daily	3449	(5)	217	(141–485)		378	(11)	316	(9)	2755	(80)	
	Missing	372	(0.6)	236	(148–500)		63	(17)	64	(17)	245	(66)	
Alcohol consumption in units/week	No alcohol	58659	(89)	314	(167–559)		14448	(25)	11528	(20)	32683	(56)	
	<0.5	6155	(9)	311	(170–556)	0.21	1326	(22)	1164	(19)	3665	(60)	<0.0001
	≥0.5	1200	(2)	286	(167–531)		249	(21)	185	(15)	766	(64)	
Partners with income of >300,000	0	18364	(28)	279	(161–540)	<0.0001	3176	(17)	2904	(16)	12284	(67)	
	1	27212	(41)	309	(166–557)		6460	(24)	5451	(20)	15301	(56)	<0.0001
	2	18608	(28)	360	(178–579)		6130	(33)	4247	(23)	8231	(44)	
NOK/year	Missing	1830	(3)	264	(158–533)		257	(14)	275	(15)	1298	(71)	
Baby’s sex	Male	33791	(51)	309	(166–558)	0.07	8190	(24)	6525	(19)	19076	(57)	0.4
	Female	32223	(49)	316	(169–559)		7833	(24)	6352	(20)	18038	(56)	
Tertiles of energy intake in MJ	1	22005	(33)	272	(127–525)		5577	(25)	4338	(20)	12090	(55)	
	2	22005	(33)	317	(166–561)	<0.0001	5511	(25)	4485	(20)	12009	(55)	<0.0001
	3	22004	(33)	344	(211–600)		4935	(22)	4054	(18)	13015	(59)	

Amount of total daily folate intake (FFQ data) and initiation of preconceptional folic acid supplementation (Q1 data) according to maternal characteristics, from 66,014 participants in the Norwegian Mother and Child Cohort Study (2002 – 2009).

^1^p-value, estimated with Kruskal-Wallis test.

^2^p-value, estimated with Pearson’s chi-squared test.

**Figure 1:**
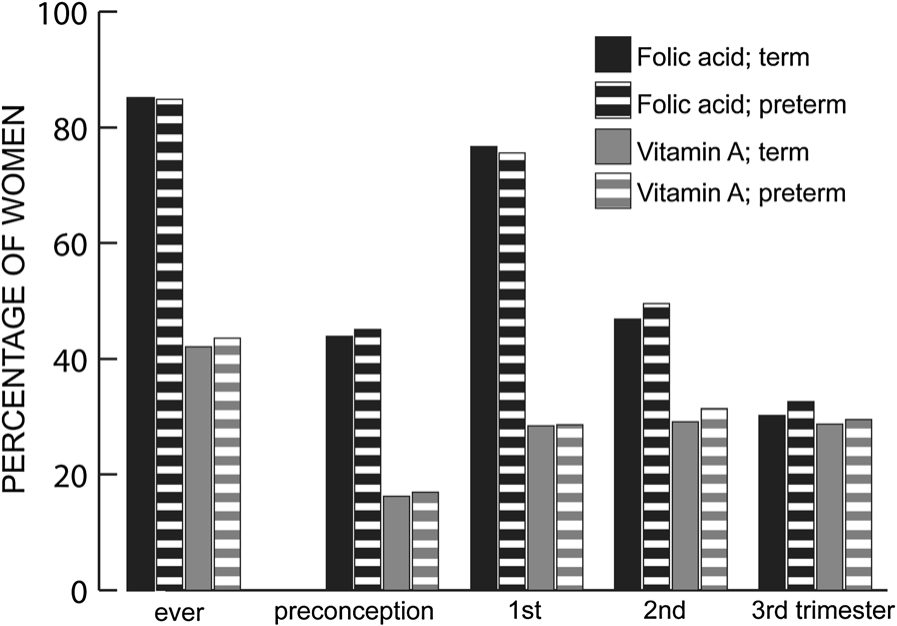
**Prevalence of folic acid and vitamin A supplementation during pregnancy.** Prevalence of folic acid and vitamin A supplementation during pregnancy (Q1 and Q3 data) in women with spontaneous non-preterm (37^+0^-42^+6^ weeks, n = 51,141) or preterm delivery (22^+0^-36^+6^ weeks, n = 1,755) among 66,014 participants in the Norwegian Mother and Child Cohort Study (2002 – 2009).

Figure 1 illustrates the pattern of folic acid supplementation in the study population compared to vitamin A supplementation (as a proxy for multivitamin consumption) over the course of pregnancy. 85% of all women in the study reported folic acid supplementation at some point before and/or during pregnancy (Figure 1). While 44% initiated folic acid supplementation prior to conception, nearly 77% used supplements containing folic acid in the first trimester with decreasing use towards the end of pregnancy. At the same time, vitamin A supplementation was much more stable over the whole length of the pregnancy. The amount of folic acid supplementation varied considerably, with only 2 women consuming folic acid amounts of >5000 μg/d, 610 consuming >1000 μg/d (1%), 6,834 consuming >500 μg/d (10%), 10,681 consuming >400 μg/d (16%), 27,183 consuming >200 μg/d (41%) and 34,234 consuming >100 μg/d (52%). Among supplement users, the median daily folic acid supplementation was 400 μg/d (interquartile range IQR 200–429). As presented in Table 2, folic acid from supplements was the main folate source in supplement users, while the main source was dietary folate in the whole population. There is no mandatory folate fortification of foods in Norway, and only 3% (n = 1,946, not adjusted for bioavailability) of the study population reached the Nordic Nutrition Recommendation of 500 μg/d with their dietary folate intake. Of the study participants, 44% (n = 28,959, not adjusted for bioavailability) achieved the recommended levels with their total folate intake.

**Table 2:** **Folate intake from diet and supplements**

	**All**		**Spontaneous delivery 37** ^**+0**^ **- 42** ^**+6**^		**Spontaneous PTD** **22** ^**+0**^ **-36** ^**+6**^	
**Folate (μg/d)**	**Median**	**IQR**	**Median**	**IQR**	**Median**	**IQR**
**All women**	n = 66014		n = 51141		n = 1755	
Diet	157	(126–196)	157	(126–196)	157	(126–196)
Supplements	143	(0–400)	143	(0–400)	171	(0–400)
Total intake	313	(167–558)	312	(167–558)	322	(167–568)
**Supplement users**	n = 39600		n = 30684		n = 1056	
Diet	158	(127–197)	158	(127–197)	159	(128–198)
Supplements	400	(200–429)	400	(200–429)	400	(200–457)
Total intake	530	(355–636)	530	(355–634)	541	(369–651)

Folic acid supplementation, dietary and total folate intake during the first half of pregnancy (FFQ data) for all 66,014 participants, as well as in folic acid supplement users (n = 39,600), from the Norwegian Mother and Child Cohort Study (2002–2009).

### Folate intake from different sources and risk for spontaneous PTD

The median gestational age in this study population was 281 days (IQR 274–287), with 2,906 (4%) cases of PTD and 1,755 cases of spontaneous PTD (2.7%). Of these, 334 babies were delivered before 34^+0^ weeks of gestation (0.5%). Thus the overall PTD rate in our study population is approximately half of the PTD rate in the general Norwegian population as we excluded risk-pregnancies due to maternal disease as well as multiple gestations and intrauterine death.

There was no significant association between the amount of folate intake from the diet or supplements with the risk of spontaneous PTD (Table 3). As 95% CIs were very narrow, we performed the same analysis in deciles of total folate intake as well with a categorized variable according to deciles of total folate intake with no change of results (data not shown). Likewise, no association was found when the total folate intake was categorized according to the former WHO recommendation for women (>170 μg/d), the current Nordic Nutrition Recommendations for pregnant women (>500 μg/d) and the tolerable upper limit (for folic acid supplementation: <1000 μg/d) (Additional file 1: Table S1). Cox regression for the sub-groups of early and late spontaneous PTD did not reveal any significant associations between the folate intake and pregnancy outcome (not shown). Testing the proportional hazards assumptions revealed a slight misfit for the parity variable. Therefore all analyses were also run with stratified Cox regression using parity as strata. The changes from the unstratified analyses were however marginal and did not change any pattern or results.

**Table 3:** **Folate intake from different sources and risk of spontaneous preterm delivery (PTD)**

	**Unadjusted**			**Adjusted** ^**2**^		
**Folate (μg/d)**	**HR** ^**1**^	**(95% CI)**	**p**	**HR** ^**1**^	**(95% CI)**	**p**
Diet	1.00	(0.61; 1.65)	0.61	1.00	(0.61; 1.65)	0.54
Supplements	1.00	(1.00; 1.00)	0.25	1.00	(1.00; 1.00)	0.53
Total intake	1.00	(1.00; 1.00)	0.22	1.00	(1.00; 1.00)	0.46

Amount of folic acid supplementation, dietary and total folate intake (FFQ data) and hazard ratios for spontaneous PTD (22^+0^-36^+6^ weeks, n = 1,755). Cox regression for 66,014 participants in the Norwegian Mother and Child Cohort Study (2002–2009). Iatrogenic deliveries have been censored in the regression model.

^1^HR per 500 μg extra folate/d.

^2^Cox regression, adjusted for maternal age, prepregnancy BMI, parity, history of PTD and spontaneous abortion, child’s sex, smoking habits and alcohol consumption during pregnancy, maternal education, marital status, household income, energy intake. Mutual adjustment for dietary and supplemental folate intake.

### Initiation of folic acid supplementation and risk for spontaneous PTD

The initiation of folic acid supplementation more than 8 weeks preconception was associated with increased risk of spontaneous PTD overall also after adjusting for potential confounders (Table 4 and Additional file 2: Figure S1a; hazard ratio HR 1.19; confidence interval CI 1.05-1.34). The initiation of supplementation more than 8 weeks before conception was significantly associated with early (<34^+0^ weeks) but not late spontaneous PTD (Additional file 3: Figure S1b). After stratification for the total dietary folate intake from foods, the initiation of folic acid supplementation more than 8 weeks before conception was significantly associated with an increased risk of spontaneous PTD for those women with low dietary folate intakes (adjusted HR 1.18; 95% CI 1.05-1.32). The same association was found in the subgroup of early (adjusted HR 1.60; 95% CI 1.24-2.10) but not late spontaneous PTD (Additional file 4: Table S2).

**Table 4:** **Initiation of preconceptional folic acid supplementation and risk of spontaneous preterm delivery (sPTD)**

**sPTD**	**Initiation of folic acid supplementation**		**Unadjusted**			**Adjusted** ^**1**^			**Adjusted** ^**2**^		
		**n**	**HR**	**(CI)**	**p**	**HR**	**(CI)**	**p**	**HR**	**(CI)**	**p**
All	No	964	1			1			1		
	0-8 w preconception	313	0.93	(0.82; 1.06)	0.28	0.99	(0.87; 1.13)	0.89	1.01	(0.88; 1.16)	0.88
	>8 w preconception	478	1.15	(1.03; 1.29)	0.01	1.18	(1.05; 1.32)	0.005	1.20	(1.06; 1.36)	0.004
Early	No	169	1			1			1		
	0-8 w preconception	57	0.97	(0.72; 1.31)	0.84	1.07	(0.79; 1.45)	0.67	1.04	(0.76; 1.43)	0.81
	>8 w preconception	108	1.48	(1.16; 1.89)	0.001	1.60	(1.24; 2.06)	<0.001	1.55	(1.18; 2.05)	0.002
Late	No	795	1			1			1		
	0-8 w preconception	256	0.92	(0.80; 1.06)	0.23	0.97	(0.84; 1.11)	0.63	0.99	(0.86; 1.15)	0.93
	>8 w preconception	370	1.08	(0.96; 1.22)	0.22	1.09	(0.96; 1.24)	0.19	1.12	(0.98; 1.29)	0.11

Initiation of preconceptional folic acid supplementation (Q1 data) and hazard ratios (HR) for spontaneous PTD (n = 1,755 for 22^+0^-36^+6^ weeks, n = 334 for early (22^+0^-33^+6^ weeks), n = 1,421 for late (34^+0^-36^+6^ weeks)). Cox regression for 66,014 participants in the Norwegian Mother and Child Cohort Study (2002 – 2009). Iatrogenic deliveries have been censored in the regression model.

^1^Cox regression, adjusted for maternal age, prepregnancy BMI, parity, history of PTD and spontaneous abortion, child’s sex, smoking habits and alcohol consumption during pregnancy, maternal education, marital status, household income, energy intake and dietary folate intake.

^2^Adjustment as above as well as for first-trimester folic acid supplementation and preconceptional and first-trimester vitamin A supplementation.

A history of earlier adverse pregnancy outcome could be a motive for the early initiation of folic acid supplementation in subsequent pregnancies. However, the analysis of the subgroup of women being pregnant the first time (n = 25,281, 38%) showed the same overall results for the early initiation of folic acid supplementation (n = 815 cases of spontaneous PTD, adjusted HR 1.30; CI 1.10-1.53).

The early initiation of folic acid supplementation could characterize women that planned a pregnancy but did not become pregnant during the first months, thus including a subgroup of sub-fertile women [37]. In MoBa, the women were asked to report the number of months with regular intercourse without contraception before becoming pregnant, and these data were classified as follows: <1 month (n = 13,128, 20%), 1–2 months (n = 15,142, 23%) and >2 months (n = 22,702, 34%). Stratification for this variable still showed increased HRs for the early initiation of folic acid supplementation in the subgroup that became pregnant within the first month (adjusted HR 1.58; CI 1.17-2.12), the HRs for the small subgroup with early PTD (n = 68) was not significant (adjusted HR 1.78; CI 0.98-3.23).

### Timing of folic acid supplementation and risk for spontaneous PTD

The time of folic acid supplementation was represented by four variables corresponding to the following periods: 26–9 weeks before conception, 0–8 weeks before conception, first trimester and second trimester. If the Cox regression included all of the confounders and the outcome of spontaneous PTD was analyzed, the prediction of the model improved after introducing all four folic acid supplementation variables (p = 0.006). Folic acid supplement use more than 8 weeks preconception was associated with an increased HR for spontaneous PTD, even after adjusting for the supplementation at all other time points (Table 5).

**Table 5:** **Timing of folic acid supplementation and risk of spontaneous preterm delivery (PTD)**

	**Time of folic acid supplementation**	**sPTD**	**Unadjusted**			**Adjusted** ^**1**^		
		**n**	**HR**	**(CI)**	**p**	**HR**	**(CI)**	**p**
All	>8 w preconception	478	1.17	(1.06; 1.30)	0.003	1.17	(1.04; 1.32)	0.01
	0-8 w preconception	648	1.01	(0.92; 1.12)	0.80	1.01	(0.90; 1.13)	0.84
	1st trimester	1326	0.94	(0.84; 1.05)	0.26	0.89	(0.79; 1.00)	0.05
	2nd trimester	869	1.10	(1.00; 1.21)	0.04	1.08	(0.98; 1.19)	0.11
Early	>8 w preconception	108	1.49	(1.19; 1.88)	0.001	1.51	(1.16; 1.97)	0.002
	0-8 w preconception	133	1.15	(0.92; 1.43)	0.23	1.03	(0.80; 1.34)	0.81
	1st trimester	262	1.11	(0.85; 1.44)	0.45	0.99	(0.74; 1.32)	0.95
	2nd trimester	173	1.21	(0.97; 1.49)	0.09	1.12	(0.89; 1.39)	0.34
Late	>8 w preconception	370	1.10	(0.98; 1.24)	0.10	1.10	(0.97; 1.26)	0.15
	0-8 w preconception	515	0.98	(0.88; 1.09)	0.69	1.00	(0.88; 1.13)	0.94
	1st trimester	1064	0.91	(0.81; 1.03)	0.13	0.87	(0.76; 1.00)	0.04
	2nd trimester	696	1.08	(0.98; 1.20)	0.13	1.08	(0.97; 1.20)	0.17

Folic acid supplementation at different times (Q1 and Q3 data) and hazard ratios (HR) for spontaneous PTD (n = 1.755 for 22^+0^-36^+6^ weeks, n = 334 for early (22^+0^-33^+6^ weeks), n = 1,421 for late (34^+0^-36^+6^ weeks)) as compared to no intake at that time of pregnancy. Cox regression for 66,014 participants in the Norwegian Mother and Child Cohort Study (2002 – 2009). Iatrogenic deliveries have been censored in the regression model.

^1^Cox regression, adjusted for maternal age, prepregnancy BMI, parity, history of PTD and spontaneous abortion, child’s sex, smoking habits and alcohol consumption during pregnancy, maternal education, marital status, household income, energy intake and dietary folate intake. Mutual adjustment for folic acid supplementation at other time points.

## Discussion

In this large prospective national birth cohort, we did not find any statistically significant association of the amount of folate intake from the diet or supplements with spontaneous PTD. Folic acid supplementation starting more than 8 weeks before conception was associated with an increased HR for spontaneous PTD.

When interpreting the results, the selection of the study population has to be kept in mind: risk-pregnancies due to maternal disease and multiple gestations have been excluded from the analysis so that we do not know if there is any association of the amount of folate intake from diet or supplements with spontaneous PTD in the excluded high risk pregnancies.

Our results, demonstrating no significant protective effect of the maternal folate intake or folic acid supplementation on the spontaneous PTD risk, support a number of earlier observational studies [38-43] and RCTs [6,44,45]. A reanalysis of a previous Cochrane review, and one of the largest RCTs, a recent meta-analysis of all RCTs published to date as well as the most recent Cochrane review based on 3 available trials, showed no association of the maternal folate status with the gestational length or the risk of PTD [23-25]. Extensive supplementation with multivitamins with a major folic acid component was associated with an increased risk of PTD in a study by Alwan et al. [38]. When analyzing data from the US National Birth Defects Study, Shaw et al. found a lower risk of PTD for women starting supplementation during pregnancy as compared to preconceptional supplementation start [43].

However, results are conflicting as summarized in the review by Mantovani et al. [27]. A protective effect of folic acid supplementation was supported by a modest reduction in the PTD rate after the introduction of folate fortification of foods [9]. Some recent observational studies have found that folic acid supplementation reduces the risk of PTD [20-22]. In some cases, this association was found for preconceptional folic acid supplementation for 1 year or longer [21] or third-trimester folic acid supplementation [22], raising questions about extended supplementation schemes compared to the NTD prevention scheme. Folic acid supplementation more than a 1 year before becoming pregnant is not registered in MoBa, but data for the earliest interval (26–9 weeks before conception) suggest an adverse effect of longtime folic acid supplementation on the risk of spontaneous PTD. Though third trimester folic acid supplementation is registered in MoBa, we chose to not include third trimester supplementation as the early PTDs already have occurred at different time points during this period, so that we were not able to retrace the findings by Czeizel et al. [22].

One possible explanation for these conflicting results could be the dosage of folic acid. While most of the studies finding an association with gestational length or PTD were based on high doses of folic acid (≥5000 μg/d [20,22,46], ≥2500 μg/d [22,47] and ≥500 μg/d [48-50]), only 2 women in our study population consumed as much as 5000 μg/d of supplemental folic acid, while only 10% consumed >500 μg/d and 16% consumed >400 μg/d. However, the Hungarian RCT, one of the biggest performed so far, did not find any effect of a high dosage of 800 μg/d of periconceptional folic acid supplementation on PTD [6]. Unfortunately, the folic acid dosage was not indicated in all of the studies [21,37].

The assessment of folate intake from supplementation alone or when studying populations with different dietary folate intakes are additional factors compromising comparability between studies. While recent US studies are performed against the background of mandatory folate fortification of food [21,43], other studies have examined supplementation effects in folate-deficient populations [46]. Few studies have assessed the effects of both dietary folate and folic acid supplementation separately [43] or combined [20,49,51], and adjustments for bioavailability are rare. In this Norwegian study population, only 3% of the participants reached the Nordic Nutrition Recommendation of 500 μg/d with their dietary folate intake, and 44% of the participants achieved the recommended level with their dietary folate and supplemental folic acid intake. After stratification for dietary folate intake, the early initiation of folic acid supplementation was significantly associated with spontaneous PTD in the subgroup of women with low but not high dietary folate intakes. There were no significant associations between high total folate intakes and PTD risk in the MoBa study population.

In observational studies, confounding is always an issue when assessing the association of a single environmental factor with a complex outcome like PTD. For example, it is well established that women with high levels of education, privileged socioeconomic status and healthier overall diets are more likely to use supplements during pregnancy [52-54] and less likely to experience PTD than women without these characteristics. Some observational studies failed to adjust for these confounders, and the effect attributed to folic acid supplementation might in fact be confounded by overall health and lifestyle behaviors. While the strength of the significance was moderate, the association with the early onset of folic acid supplementation in the current study remained significant even after adjustment for maternal characteristics such as socioeconomic and life-style parameters as well as obstetric anamnesis.

Associations with the early start of supplementation should be studied with particular caution. The early start of folic acid supplementation might partially identify a group of women with a history of adverse pregnancy outcomes who want to optimize conditions for their current pregnancy. As presented in Table 1, women who had previously experienced spontaneous abortions were more likely to initiate folic acid supplementation early in their subsequent pregnancies. However, the same association of the early initiation of folic acid supplementation and spontaneous PTD was found in women being pregnant the first time. Folic acid supplementation starting more than 8 weeks prior to conception might characterize women who planned a pregnancy but did not become pregnant during their first 2 cycles, thus constituting a subgroup of women with suboptimal fertility [37]. The same association of the early start of supplementation was found in the group of women that became pregnant within the first month. Women who choose to start early with folic acid supplementation might be distinguished by some other characteristic that could be the causal link to spontaneous preterm delivery so that we cannot exclude confounding.

In addition to the amount of folic acid, the composition of supplements is another point of discussion. In some countries like Greece and Norway, commonly used supplements contain folic acid and/or iron only [20]. In other countries, folic acid is mainly consumed in the form of multivitamins, making it difficult to differentiate the effects of multivitamin use and folic acid supplementation [21,37,43,50]. Vitamins other than folic acid might explain the association between multivitamin use and PTD. Catov et al. found that in the Danish birth cohort, multivitamin use was associated with modestly decreased PTD rates, while there was no association with folic acid supplementation [39]. As seen from Figure 1, vitamin A consumption (as a proxy for multivitamin supplementation) differed considerably from folic acid supplementation. However, the MoBa FFQ allowed us to calculate folic acid separately from other supplements, and adjusting for vitamin A consumption did not change the results for preconceptional folic acid supplementation.

Apart from the amount, timing and composition of folate exposure, differences in the definition of pregnancy outcomes hinder comparability. Most studies defined PTD as delivery at <37^+0^ weeks of gestation without indicating the range of gestational age. This information might be important, especially if the risk of early PTD is found to be associated with folate status, as suggested by this study and that of Bukowski et al. [21]. Although PTD is a heterogeneous pregnancy outcome with distinct etiologies for different subgroups [26], not all studies analyzed clearly defined subgroups such as spontaneous PTD [20,21,39,42,49,55].

### Strengths and weaknesses

With a sample size of 66,014 pregnancies, this was a well-powered study for investigating the association of folate intake and spontaneous PTD. Due to the large study sample, there were 1,755 cases defined as spontaneous PTD and 334 and 1,421 cases in the subgroups of early and late spontaneous PTD, respectively. The estimation of the gestational length by the second-trimester ultrasounds and the definition of a clear PTD phenotype – spontaneous onset of delivery between 22^+0^ and 36^+6^ weeks of gestation - distinguish this study.

The MoBa participation rate is 38.5%, and a demographic comparison with the MBRN in 2002 showed that single women and women <25 y of age are underrepresented in MoBa. Regarding PTD (7.2% in MoBa and 7.7% in MBRN), the differences are minor, and even the sub-group composition is similar to the distribution in the total population, with spontaneous PTD accounting for 42% of all PTD [15]. Although the low participation in MoBa influences prevalence estimates somewhat, such non-representativeness does not appear to affect exposure–outcome associations [56,57].

The assessment of folate from both the diet and supplements is a major strength of this study. Although all dietary assessment methods have limitations, the MoBa FFQ has been extensively validated in a sub-population of 119 MoBa participants using a 4-day weighed food diary and biological markers in the blood and urine as reference measures [58]. The dietary supplement use was evaluated specifically. The total folate intake by the FFQ showed good agreement with the folate intake detailed by the food diary and was significantly reflected by the serum folate concentrations [33]. In a subsample of an earlier MoBa version (2934 singleton pregnancies), Nilsen et al. did not find any significant associations of dietary folate intake, folic acid supplementation or plasma folate with PTD. This study also reported good agreement between the folate intake (dietary and supplements) by the MoBa FFQ and plasma folate concentration (r = 0.44, CI: 0.41-0.47) [40]. As the relevant window of susceptibility for folate effects regarding pregnancy outcomes other than NTD is not yet known, the assessment of folate intake at different time points is a further strength of this study. The prospective design ensured that the women’s answers were not influenced by their knowledge of pregnancy outcomes.

## Conclusions

The overall amount of dietary folate and supplemental folic acid intake (during the first half of pregnancy) in 66,014 singleton pregnancies from the Norwegian Mother and Child Cohort Study was not associated with decreased or increased risk of spontaneous PTD, at least not at the relatively low intake levels of dietary folate (median 157 μg/d corrected for bio-availability, uncorrected 262 μg/d) and supplemental folic acid (median 143 μg/d) in this healthy study population.

The initiation of folic acid supplementation more than 8 weeks prior to conception was associated with an increased risk for overall and early spontaneous PTD in both the overall analyses and in the strata of women with low dietary folate intake.

Even if MoBa allows adjustment for a variety of confounders, the presence of residual confounding cannot be ruled out. Furthermore, our results require careful investigation regarding dosage and timing of folic acid supplementation, such as in the form of an RCT, before discussing a change of the current guidelines.

## Additional files

Additional file 1: Table S1. Folate intake according to official recommendations and risk of spontaneous preterm delivery (PTD). Additional file 2: Figure S1a. Initiation of preconceptional folic acid supplementation and risk of spontaneous PTD. Initiation of preconceptional folic acid supplementation (Q1 data) and cumulative risk of spontaneous PTD (22^+0^-36^+6^ weeks, n = 1,755). Cox regression for 66,014 participants in the Norwegian Mother and Child Cohort Study (2002 – 2009), adjusted for maternal age, prepregnancy BMI, parity, history of PTD and spontaneous abortion, child’s sex, smoking habits and alcohol consumption during pregnancy, maternal education, marital status, household income, energy intake and dietary folate intake. Iatrogenic deliveries have been censored in the regression model. Additional file 3: Figure S1b. Initiation of preconceptional folic acid supplementation and risk of early spontaneous PTD. Initiation of preconceptional folic acid supplementation (Q1 data) and cumulative risk of early spontaneous PTD (22^+0^-33^+6^ weeks, n = 334). Cox regression for 66,014 participants in the Norwegian Mother and Child Cohort Study (2002 – 2009), adjusted for maternal age, prepregnancy BMI, parity, history of PTD and spontaneous abortion, child’s sex, smoking habits and alcohol consumption during pregnancy, maternal education, marital status, household income, energy intake and dietary folate intake. Iatrogenic deliveries have been censored in the regression model. Additional file 4: Table S2. Initiation of preconceptional folic acid supplementation and risk of spontaneous preterm delivery (sPTD), depending on dietary folate intake.

## Abbreviations

BMI: Body mass index
CI: Confidence interval
FFQ: Food frequency questionnaire
HR: Hazard ratio
IQR: Interquartile range
MoBa: The Norwegian Mother and Child Cohort Study
MBRN: Medical birth registry of Norway
NOK: Norwegian crowns, currency
NTD: Neural tube defect
PTD: Preterm delivery
Q1: Q3, Questionnaire 1, 3
RCT: Randomized controlled trial
WHO: World health organization
