# Supplementary material for: Folic acid supplementation, dietary folate intake during pregnancy and risk for spontaneous preterm delivery: a prospective observational cohort study
Source: BMC Pregnancy Childbirth. 2014 Nov 2;14:375. doi: 10.1186/s12884-014-0375-1 (PMC4240839; doi:10.1186/s12884-014-0375-1)
Supplement: Additional file 1: Table S1. — Folate intake according to official recommendations and risk of spontaneous preterm delivery (PTD). [file 12884_2014_375_MOESM1_ESM.doc]

### Additional 1: Table S1. Folate intake according to official recommendations and risk of spontaneous preterm delivery (PTD).

Amount of total daily folate intake (FFQ data) and hazard ratios for spontaneous PTD (22^+0^-36^+6^ weeks, n=1,755). Cox regression for 66,014 participants in the Norwegian Mother and Child Cohort Study (2002-2009). Total folate intake is categorized according to the former WHO recommendations for women (>170 μg/d), the current Nordic Nutrition Recommendations for pregnant women (>500 μg/d) and the tolerable upper limit (<1000 µg/d). Iatrogenic deliveries have been censored in the regression model.

| Folate intake (µg/d) | sPTD | unadjusted | | | adjusted^1^ | | |
| --- | --- | --- | --- | --- | --- | --- | --- |
|  | n | HR | (CI) | p | HR | (CI) | p |
| <170 | 459 | 1 |  |  | 1 |  |  |
| 170-499 | 669 | 0.95 | (0.84; 1.07) | 0.38 | 0.94 | (0.83; 1.07) | 0.33 |
| 500-1000 | 583 | 1.02 | (0.90; 1.15) | 0.81 | 0.99 | (0.88; 1.13) | 0.93 |
| >1000 | 44 | 1.15 | (0.85; 1.57) | 0.37 | 1.07 | (0.78; 1.46) | 0.68 |

^1^ Cox regression, adjusted for maternal age, prepregnancy BMI, parity, history of PTD and spontaneous abortion, child’s sex, smoking habits and alcohol consumption during pregnancy, maternal education, marital status, household income, energy intake.
