# Supplementary figures and images for: Folic acid supplementation, dietary folate intake during pregnancy and risk for spontaneous preterm delivery: a prospective observational cohort study
Source: BMC Pregnancy Childbirth. 2014 Nov 2;14:375. doi: 10.1186/s12884-014-0375-1 (PMC4240839; doi:10.1186/s12884-014-0375-1)

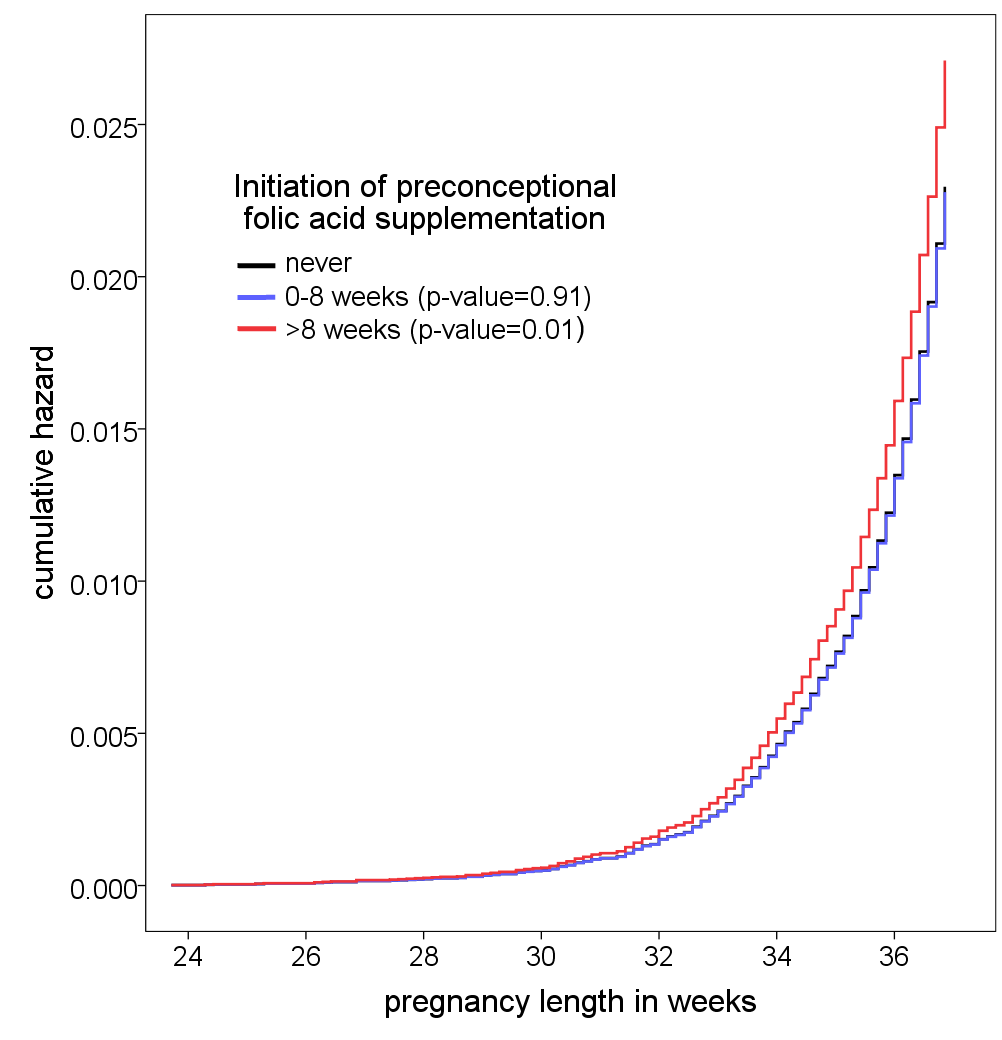

Supplement: Additional file 2: Figure S1a. — Initiation of preconceptional folic acid supplementation and risk of spontaneous PTD. Initiation of preconceptional folic acid supplementation (Q1 data) and cumulative risk of spontaneous PTD (22+0-36+6 weeks, n = 1,755). Cox regression for 66,014 participants in the Norwegian Mother and Child Cohort Study (2002 – 2009), adjusted for maternal age, prepregnancy BMI, parity, history of PTD and spontaneous abortion, child’s sex, smoking habits and alcohol consumption during pregnancy, maternal education, marital status, household income, energy intake and dietary folate intake. Iatrogenic deliveries have been censored in the regression model. [file 12884_2014_375_MOESM2_ESM.tiff]

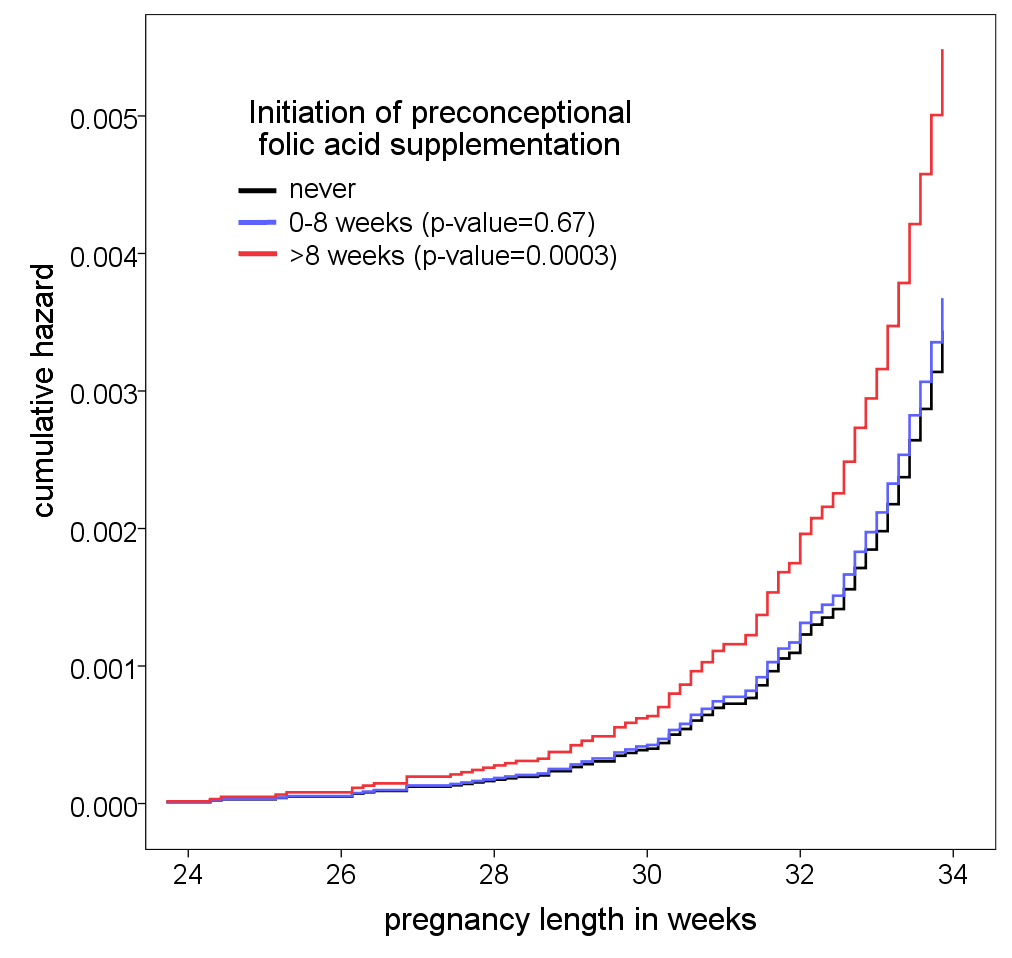

Supplement: Additional file 3: Figure S1b. — Initiation of preconceptional folic acid supplementation and risk of early spontaneous PTD. Initiation of preconceptional folic acid supplementation (Q1 data) and cumulative risk of early spontaneous PTD (22+0-33+6 weeks, n = 334). Cox regression for 66,014 participants in the Norwegian Mother and Child Cohort Study (2002 – 2009), adjusted for maternal age, prepregnancy BMI, parity, history of PTD and spontaneous abortion, child’s sex, smoking habits and alcohol consumption during pregnancy, maternal education, marital status, household income, energy intake and dietary folate intake. Iatrogenic deliveries have been censored in the regression model. [file 12884_2014_375_MOESM3_ESM.tiff]
