# Supplementary material for: Folic acid supplementation, dietary folate intake during pregnancy and risk for spontaneous preterm delivery: a prospective observational cohort study
Source: BMC Pregnancy Childbirth. 2014 Nov 2;14:375. doi: 10.1186/s12884-014-0375-1 (PMC4240839; doi:10.1186/s12884-014-0375-1)
Supplement: Additional file 4: Table S2. — Initiation of preconceptional folic acid supplementation and risk of spontaneous preterm delivery (sPTD), depending on dietary folate intake. [file 12884_2014_375_MOESM4_ESM.doc]

### Additional 4: Table S2. Initiation of preconceptional folic acid supplementation and risk of spontaneous preterm delivery (sPTD), depending on dietary folate intake.

Initiation of preconceptional folic acid supplementation (Q1 data) and hazard ratios for spontaneous PTD (n=1,755 for 22^+0^-36^+6^ weeks, n=334 for early (22^+0^-33^+6^ week), n=1,421 for late (34^+0^-36^+6^ weeks)) stratified for dietary folate intake. Cox regression for 66,014 participants in the Norwegian Mother and Child Cohort Study (2002 – 2009). Iatrogenic deliveries have been censored in the regression model.

| sPTD | Dietary | Initiation of folic acid |  | unadjusted | | | adjusted1 | | | adjusted2 | | |
| --- | --- | --- | --- | --- | --- | --- | --- | --- | --- | --- | --- | --- |
|  | folate (µg/d) | supplementation | n | HR | (CI) | p | HR | (CI) | p | HR | (CI) | p |
|  | <170 | No | 583 | 1 |  |  | 1 |  |  | 1 |  |  |
|  |  | 0-8 w preconception | 196 | 0.99 | (0.84; 1.16) | 0.87 | 1.06 | (0.90; 1.25) | 0.51 | 1.09 | (0.92; 1.29) | 0.33 |
| all |  | >8 w preconception | 286 | 1.18 | (1.02; 1.36) | 0.02 | 1.21 | (1.05; 1.41) | 0.01 | 1.25 | (1.07; 1.47) | 0.01 |
|  | ≥170 | No | 381 | 1 |  |  | 1 |  |  | 1 |  |  |
|  |  | 0-8 w preconception | 117 | 0.85 | (0.69; 1.05) | 0.14 | 0.91 | (0.73; 1.12) | 0.36 | 0.91 | (0.73; 1.13) | 0.39 |
|  |  | >8 w preconception | 192 | 1.11 | (0.94; 1.32) | 0.23 | 1.14 | (0.95; 1.37) | 0.16 | 1.14 | (0.93; 1.38) | 0.21 |
|  | <170 | No | 111 | 1 |  |  | 1 |  |  | 1 |  |  |
|  |  | 0-8 w preconception | 39 | 1.03 | (0.72; 1.49) | 0.86 | 1.18 | (0.81; 1.71) | 0.39 | 1.18 | (0.80; 1.73) | 0.41 |
| early |  | >8 w preconception | 67 | 1.45 | (1.07; 1.96) | 0.02 | 1.64 | (1.19; 2.26) | 0.002 | 1.65 | (1.17; 2.34) | 0.01 |
|  | ≥170 | No | 58 | 1 |  |  | 1 |  |  | 1 |  |  |
|  |  | 0-8 w preconception | 18 | 0.87 | (0.51; 1.60) | 0.59 | 0.92 | (0.54; 1.58) | 0.76 | 0.85 | (0.49; 1.48) | 0.57 |
|  |  | >8 w preconception | 41 | 1.56 | (1.05; 2.52) | 0.03 | 1.57 | (1.03; 2.39) | 0.04 | 1.42 | (0.90; 2.26) | 0.14 |
|  | <170 | No | 472 | 1 |  |  | 1 |  |  | 1 |  |  |
|  |  | 0-8 w preconception | 157 | 0.97 | (0.81; 1.16) | 0.73 | 1.02 | (0.85; 1.23) | 0.83 | 1.06 | (0.87; 1.28) | 0.58 |
| late |  | >8 w preconception | 219 | 1.11 | (0.95; 1.31) | 0.20 | 1.11 | (0.94; 1.32) | 0.21 | 1.15 | (0.96; 1.38) | 0.12 |
|  | ≥170 | No | 323 | 1 |  |  | 1 |  |  | 1 |  |  |
|  |  | 0-8 w preconception | 99 | 0.85 | (0.67; 1.06) | 0.14 | 0.90 | (0.71; 1.13) | 0.35 | 0.91 | (0.72; 1.16) | 0.45 |
|  |  | >8 w preconception | 151 | 1.04 | (0.85; 1.26) | 0.72 | 1.06 | (0.87; 1.30) | 0.55 | 1.08 | (0.87; 1.34) | 0.50 |

^1^ Cox regression. adjusted for maternal age, prepregnancy BMI, parity, history of PTD and spontaneous abortion, child’s sex, smoking habits and alcohol consumption during pregnancy, maternal education, marital status, household income, energy intake and dietary folate intake.
^2^ Adjustment as above as well as for first trimester folic acid supplementation and pre-conceptional and first trimester vitamin A supplementation.
